# Biosocial Conservation: Integrating Biological and Ethnographic Methods to Study Human–Primate Interactions

**DOI:** 10.1007/s10764-016-9938-5

**Published:** 2016-12-17

**Authors:** Joanna M. Setchell, Emilie Fairet, Kathryn Shutt, Siân Waters, Sandra Bell

**Affiliations:** 1 Department of Anthropology, Durham University, Durham, DH1 3LE UK; 2 Behaviour, Ecology and Evolution Research (BEER) Centre, Durham University, Durham, DH1 3LE UK; 3 Sustainable Forest Management (SFM) Gabon, Libreville, Gabon; 4 Fauna & Flora International, Cambridge, CB2 3QZ UK; 5 Barbary Macaque Awareness and Conservation (BMAC), Tetouan, Morocco

**Keywords:** Cultural anthropology, Ethnobiology, Ethnoprimatology, Human–primate interactions, Interdisciplinary, Social anthropology

## Abstract

Biodiversity conservation is one of the grand challenges facing society. Many people interested in biodiversity conservation have a background in wildlife biology. However, the diverse social, cultural, political, and historical factors that influence the lives of people and wildlife can be investigated fully only by incorporating social science methods, ideally within an interdisciplinary framework. Cultural hierarchies of knowledge and the hegemony of the natural sciences create a barrier to interdisciplinary understandings. Here, we review three different projects that confront this difficulty, integrating biological and ethnographic methods to study conservation problems. The first project involved wildlife foraging on crops around a newly established national park in Gabon. Biological methods revealed the extent of crop loss, the species responsible, and an effect of field isolation, while ethnography revealed institutional and social vulnerability to foraging wildlife. The second project concerned great ape tourism in the Central African Republic. Biological methods revealed that gorilla tourism poses risks to gorillas, while ethnography revealed why people seek close proximity to gorillas. The third project focused on humans and other primates living alongside one another in Morocco. Incorporating shepherds in the coproduction of ecological knowledge about primates built trust and altered attitudes to the primates. These three case studies demonstrate how the integration of biological and social methods can help us to understand the sustainability of human–wildlife interactions, and thus promote coexistence. In each case, an integrated biosocial approach incorporating ethnographic data produced results that would not otherwise have come to light. Research that transcends conventional academic boundaries requires the openness and flexibility to move beyond one’s comfort zone to understand and acknowledge the legitimacy of “other” kinds of knowledge. It is challenging but crucial if we are to address conservation problems effectively.

## Introduction

Biodiversity conservation is inherently a “wicked” problem (Game *et al*. 2014). Wicked, in the sense of “vicious” or “tricky,” problems have no definitive formulation, no clear end-point at which the solution is found, and no true or false answers (Rittel and Webber 1973). Solutions to wicked problems have waves of consequences over an extended period of time and it is impossible to implement a trial solution without incurring consequences (Rittel and Webber 1973). Moreover, every problem is essentially unique, precluding principles of solution; causes are contested; and the analyst’s worldview is the strongest determining factor in the choice of an explanation for a problem (Rittel and Webber 1973). No wonder, then, that confronting conservation problems can be paralyzing.

Attempts to understand and resolve conservation problems, like other wicked problems, require an inclusive community of practitioners, including biological and social scientists who work together as a team and/or individuals able to integrate multiple approaches (Green *et al*. 2015). A dizzying list of disciplines is relevant to conservation, including anthropology, biology, development studies, geography, politics, psychology, education, economics, and history (Newing 2010). Many people become interested in biodiversity conservation from a background in wildlife biology (like four of the five of the authors of this article), or a deep desire to help, or rescue, animals. Although this is excellent motivation, and good educational background, it may not be enough on its own. Data on a species’ population size and distribution, life history, behavior, and ecology are necessary to determine its threat status. However, the threats to species’ survival are overwhelmingly anthropogenic (Ceballos *et al*. 2015; Chapman and Peres 2001; Cowlishaw and Dunbar 2000; Estrada *et al*. accepted; Oates 2013), meaning that conservation problems are at least as much about people as they are about animals. This realization, which has been termed an epiphany for natural scientists (Cowling 2005 cited in Balmford and Cowling 2006), makes it as important to understand the social and cultural (i.e., the anthropological) aspects of conservation as it is to collect and analyze ecological data if we are to conceive locally relevant, effective conservation strategies (Balmford and Cowling 2006; Pretty *et al*. 2009; Wolverton *et al*. 2014).

Anthropogenic influences on primates are multifaceted, including human-related variation in landscape, the nature of the human–other primate interface, diet, and predation (McKinney 2015). Understanding these relations, wherever they occur, is vital for conservation of both primates and their ecosystems (Chapman and Peres 2001; Riley and Fuentes 2011). In this article, we demonstrate the relevance and value of an integrative approach that combines biological and social science methods to the complex issues involved in conservation. We first introduce anthropological approaches to conservation and provide a brief introduction to social science methods for life scientists. We then review case studies at three sites that vary in the level and nature of anthropogenic influence (Table I). The first project involves people living around a newly established national park in Gabon, the second concerns great ape tourism in a national park in the Central African Republic, and the third focuses on primates and people living alongside one another in Morocco. We conclude with what we have learned from these studies and the challenges posed by interdisciplinary work. The interdisciplinary methods we advocate are relevant for studying primates in human-dominated landscapes (the topic of this special issue), and for studying primates more broadly, as few primates live in idealized “pristine” habitats.

**Table I:** The three case studies described in this article, with details of the anthropogenic influence at each site

Case study	Animal species	Landscape	Location	Diet of the animal species	Humans involved in the interface	Predation	
1	Elephants, ≥7 species of diurnal primate	Protected area, buffer zone, and a village bordering a protected area	Fields cleared in forest environment, good habitat connectivity (E?)	Loango National Park, Gabon	Includes anthropogenic food sources, regular crop-raiding (% of diet unknown) (F)	Primarily local people, competition for resources, variable interactions (some trapping) (?)	Human predation in the form of subsistence hunting and trapping and retaliatory killing; all known indigenous predators present (H)
2	Western lowland gorillas	Protected area	Tourist/ research area with a camp but few trails (C)	Dzanga–Sangha Reserve, Central African Republic	Completely wild-foraged diet, of adequate composition and abundance (A)	Tourists, tourism teams, and researchers; daily proximity, ranging from major (habituation attempts) to minor interactions (observation and photography of habituated animals) (E)	Human predation rare; all known indigenous predators present (A)
3	Barbary macaques	Non-protected area	Pastoral landscape shared with humans (L?)	Bouhachem forest, Northern Morocco	Mostly wild-foraged foods, with the addition of opportunistic crop-raiding (C)	Primarily local people, frequency unknown, interactions ranging from minor (proximity) to intense (predation and harassment) (?)	Human predation for pest management (O), commercial hunting for pet trade plus all known indigenous predators as well as new or domesticated predators (J, L). Also human predation for amusement

Letters in () denote MacKinney’s Anthropogenic Influence Classification System (McKinney 2015). A letter followed by ? indicates uncertainty. ? indicates a situation not included in the system

## Anthropological Approaches to Conservation

Anthropological approaches to conservation are rooted in the study of traditional knowledge systems, human–environment interactions, and cultural perspectives of the environment, or ethnobiology (Ford 2011; Newing 2010; Sillitoe and Alshawi 2014; Sillitoe *et al*. 2010). Ethnobiology addresses the fact that humans and other animals inhabit the same social and ecological landscapes: they are entangled. This challenges the Western conception of boundaries and their maintenance between humans and animals, which can obscure the far greater complexity of human–animal interactions in other settings (Knight 2003). For primates, in particular, a long history of studies of coexistence with humans has led to the interdisciplinary field of ethnoprimatology (Fuentes and Hockings 2010; Jones-Engel *et al*. 2003; Lee 2010; Loudon *et al*. 2006; Malone *et al*. 2014; Papworth *et al*. 2013; Riley 2013; Sommer 2011). Ethnoprimatology explicitly acknowledges humans as active constituents of, and immersed in, biological communities and ecosytems, in contrast to the naturalistic approach to field primatology, and brings the anthropological tools of critical reflection to bear on relations between humans and other primates (Fuentes 2012; Lee 2010; Malone *et al*. 2014).

Studies integrating quantitative biological and qualitative social data show convincingly that doing so leads to a more nuanced understanding of conservation issues than studies based on single approaches (Hill and Wallace 2012; Nekaris *et al*. 2010; Remis and Hardin 2009). For example, research integrating ecological transect data and ethnography in Dzanga Sangha Reserve in the Central African Republic demonstrates how the concept of “transvaluation”—valuing species based on their ecological, economic, and symbolic roles in human lives—moves beyond dichotomized Western vs. other ways of thinking about wildlife to recognize the diverse human communities that co-occupy a landscape with wildlife and shape its survival (Remis and Hardin 2009). The addition of market survey data in subsequent work at the same site provides an excellent example of how incorporating multiple approaches leads to a more nuanced understanding of changes in both wildlife populations and economies than each individual dataset could provide (Jost Robinson *et al*. 2011). Importantly, conclusions based on each individual dataset would be likely to lead to different policy recommendations (Jost Robinson *et al*. 2011). Similarly, a combination of quantitative data on the Convention on International Trade in Endangered Species of Wild Flora and Fauna (CITES) numbers and ethnographic methods revealed both the extent of trade in lorises (*Nycticebus* and *Loris* spp.) and the drivers of that trade, providing guidance for the development of conservation strategies (Nekaris *et al*. 2010). Finally, a combination of ecological and social science methods allowed researchers to test the efficacy of deterrents to primate crop-foraging and to understand and acknowledge farmers’ interests and perceptions of those deterrents, informing intervention strategies (Hill and Wallace 2012).

## A Brief Introduction to Social Science Methods for Life Scientists

Natural scientists who employ social science methods but are unfamiliar with their philosophical basis can misinterpret their results (Moon and Blackman 2014), making interdisciplinary training in social science, collaboration with social scientists, or both essential for a successful study. The life sciences (the branch of the natural sciences that involves the scientific study of organisms) and the social sciences have very different philosophical foundations and theoretical assumptions, types of analysis, vocabulary, and styles of writing (Evely *et al*. 2008; Moon and Blackman 2014), making interdisciplinary engagement daunting. As with any interdisciplinary endeavor, differences in methodology, epistemology, and language can lead to misunderstandings and contention. Several recent papers in conservation and ecological journals illustrate this problem and attempt to bridge this gap (Moon and Blackman 2014; Sandbrook *et al*. 2013; St. John *et al*. 2014). Here, we provide brief details of key methods, highlighting differences between the social and life sciences, as an introductory guide for primatologists.

Qualitative ethnographic methods include semistructured interviews and participant observation. Semistructured interviews are based on a prepared list of topics and questions, but allow the conversation between researcher and informant to include topics and ideas brought up as the interview progresses. They allow informants to express their own understandings in their own words (Drury *et al*. 2011). Participant observation—living among informants and joining in their activities—facilitates a deep understanding and interpretation of the meanings of people’s actions and experiences (Bernard 2006). It can illuminate a situation or context not always apparent through interviews or questionnaires alone by allowing the researcher to see what people actually do, not just how they talk about what they do or what they claim to do in questionnaires or structured interviews (Bernard 2006). Participant observation can uncover contradictions between what people say and what they do, or between what people say at different times and circumstances (Drury *et al*. 2011).

Researchers with a biological background, including most field primatologists, are rarely trained in qualitative methods. As a result, life scientists who adopt social science research methods often choose quantitative instruments, such as questionnaires, to measure people’s perceptions of wildlife (Newing 2011; Verissimo 2013; White *et al*. 2005). However, such methods may not provide the nuanced insight into the diverse social, cultural, political, and historical factors that influence human–animal relations that qualitative ethnographic data can, and may conceal rather than reveal issues pertinent to conservation practice (Goldman *et al*. 2010, 2013; Kuriyan 2002; Pratt *et al*. 2004; Satterfield *et al*. 2013). While questionnaires reflect the preoccupations and perspective of the researcher, qualitative approaches enable culturally sensitive analysis of the complex relationships among attitudes, values, and behavior as understood by the population under study. Such approaches also allow the researcher to study culturally sensitive matters or contradictions between what people say and do that might be missed in more structured approaches (Drury *et al*. 2011).

Reflecting on fieldwork can be a useful introduction to participant observation, as many primatologists spend years living alongside people from other cultures. Becoming familiar with people from different cultural orientations, and understanding their worldview, is central to the practice of participant observation. Humans have an intrinsic capacity for ethnography, which has been termed “deep hanging out” (Geertz 1998). Growing up in any society involves social learning, a process intrinsic to being human and to ethnographic enquiry. Finding ourselves in entirely novel social environments, we have an innate capacity to learn how to conduct ourselves more or less acceptably. Like the ethnographer immersed in a new social world, we figure out the answers to questions we would not even know to ask from the outside, etic, perspective (Whyte 1955).

Training in the life sciences teaches us to seek an objective representation of reality that is free from emotions and subjective interpretation. Life scientists, including many primatologists, use a hypothetico–deductive approach and focus on samples large enough to allow statistical analysis, chosen to be representative of a larger population. This positivist approach contrasts with the qualitative social sciences, which allow and take account of researcher subjectivity (Pru 1996). For example, social anthropologists use qualitative ethnographic data to interpret the meanings and motivations underlying human behavior. This method is exploratory and comparative. It deploys interviewing and participant observation to generate data that can be analyzed to reveal those understandings that particular groups of people share and act on in particular circumstances. Different groups often hold conflicting understandings of animals, or other elements of the natural world, that lead to contestation (Orlove and Brush 1996). Equally, however, groups may share some overlapping evaluations that can offer a starting point for mutuality and compromise (Orlove and Brush 1996). In place-based studies, emergent theory provides insight and understanding into local people’s realities, and can enable the development of meaningful and effective engagement with local conservation problems (Pratt *et al*. 2004). Such an approach is holistic, reflexive, and situated. In other words, it acknowledges the complexity of the entire situation and encourages a rounded perspective that includes reflection on the researchers’ own orientation to the problem, particularly the way that it may be perceived by other parties. For example, local people might be reluctant to engage in conservation measures, or may even be hostile to such projects, because they identify conservationists with government agencies or other bodies that have exercised power over them in the past or do so currently (Constant and Bell 2016; Fairet 2012; Waters 2014). The findings of such studies are specific to the people studied, and do not necessarily generalize to other peoples. These methods are challenging and confusing to life scientists, who ask how a subjective interpretation can be a valid “result.” At the same time, social scientists find that generalizing (as we do in this sentence) removes all the interesting complexity from an analysis, and criticize life scientists for being “reductionist.”

In each of the three case studies that follow, a single researcher conducted the project (each was a PhD project), mentored by one biological anthropologist (Jo Setchell) and one social anthropologist (Sandra Bell).

## Case 1: Vulnerability to Wildlife Foraging on Crops: An Interdisciplinary Investigation in Loango National Park, Gabon (Emilie Fairet)

Conflicts over conservation endeavors, whether directly between wildlife and people or between stakeholders over wildlife conservation strategies and implementation, are a major threat to the long-term survival of wildlife and to the livelihoods of subsistence communities in developing countries, particularly near protected areas (Barnes 1996; Hill *et al*. 2002; Sitati *et al*. 2005; Thirdgood *et al*. 2005; Woodroffe *et al*. 2005). Interactions between humans and wildlife that result in negative impacts for either group are often termed “human–wildlife conflict”, both in the literature and by conservation organizations. However, this term has been criticized for promoting the idea that humans and wildlife are conscious adversaries (Peterson *et al*. 2010), for distracting attention from conflicts between humans about conservation (Redpath *et al*. 2013), and for ignoring the positive aspects of human–wildlife relationships (Hill 2015; Hill, this issue).

Emilie’s experiences working for great ape habituation and conservation projects in Lopé and Loango National Parks, Gabon, led to an interest in conservation conflicts in the context of Gabon’s relatively new national parks system (announced in 2002). Specifically, her observations of the effect of wildlife foraging on crops planted by her Gabonese colleagues’ families, living in villages inside Loango National Park and in or close to the buffer zone of the park, sparked an interest in the implications of conservation for the people who live alongside the park.

**Fig. 1:**
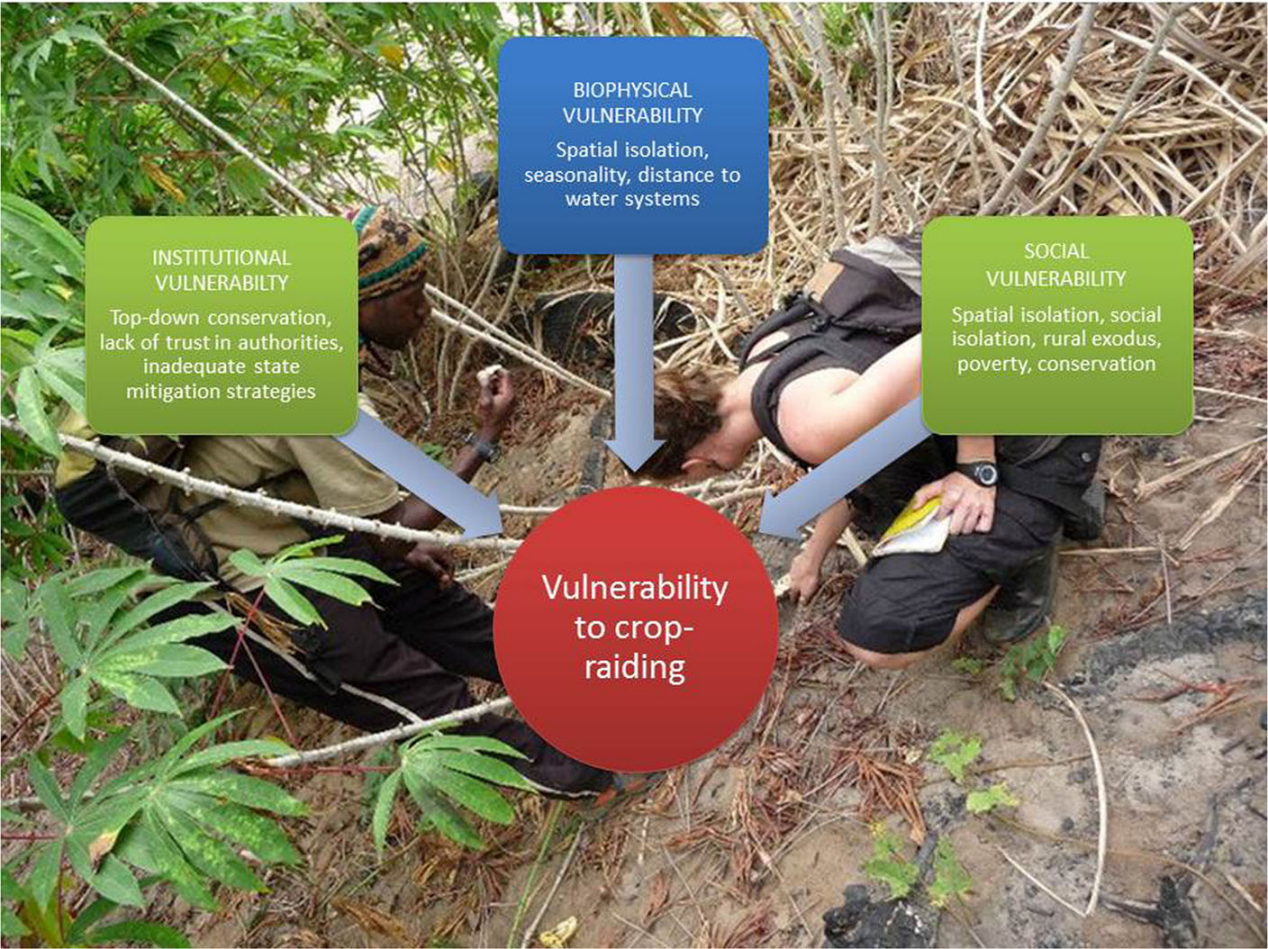
Vulnerability to wildlife foraging on crops: An interdisciplinary investigation in Loango National Park, Gabon (Emilie Fairet). Emilie and Kharl Remanda investigate signs of crop-foraging in a field near Loango National Park. Red indicates the overall focus of the study; green, use of social science methods; blue, use of natural science methods.

When wildlife forages on crops, this behavior is often termed “crop-raiding,” although the intention implied by “raiding” is inappropriate (Hill 2015). We therefore use the term “crop-foraging”. Crop-foraging is perhaps the most common and significant form of human–wildlife conflict in Africa (Naughton-Treves 1998; Sitati *et al*. 2003) and has therefore been the main focus of human–wildlife conflict studies. Crop-foraging can be investigated using a diversity of approaches. For example, studies focus on crop losses (Barnes *et al*. 2006; Chiyo *et al*. 2005; Hoare 1999; Rode *et al*. 2006; Sitati *et al*. 2005; Tweheyo *et al*. 2005), how crop-foraging affects farmers’ attitudes toward wildlife (De Boer and Baquete 1998; Gillingham and Lee 2003; Hill 1998; Hill and Webber 2010; Ogra 2009), mitigation strategies (Graham and Ochieng 2008; Hill and Wallace 2012; Osborn and Hill 2005; Osborn and Parker 2002; Sitati and Walpole 2006), the implications of crop-foraging for food security (Barirega *et al*. 2010; Hartter *et al*. 2011; Kaswamila *et al*. 2007), and animal foraging strategies (McLennan 2013; Naughton-Treves *et al*. 1998; Priston and Underdown 2009). Following a suggestion made by Naughton-Treves and colleagues (Naughton-Treves 1997; Naughton *et al*. 1999), Emilie adopted vulnerability (Adger *et al*. 2003; Birkmann 2006; Blaikie *et al*. 1994; Cannon *et al*. 2003; Carter 1997; Cutter 1996; Eakin and Luers 2006; Turner II *et al*. 2003) as a conceptual framework to integrate institutional, biophysical, and social vulnerability to crop-foraging in Loango (Fig. 1). Vulnerability science is described as helping to “understand those circumstances that put people and places at risk, and those conditions that reduce the ability of people and places to respond to environmental threats” (Cutter 2003: 6).

Emilie based herself in two remote villages in Loango National Park for more than a year. She used a combination of participant observation, an ethnographic journal, and semistructured interviews to investigate the institutional and social context of crop-foraging, and quantitative survey methods to assess the biophysical aspect of crop-foraging by medium to large mammals.

Emilie first investigated the context of conservation at her study site, and how this limits, or intensifies, conflict over wildlife. She showed that protected areas were implemented as a top-down conservation strategy in Gabon. The process excluded local populations from the decision-making processes and eroded or prevented feelings of ownership over their natural resources in local people, thus contributing to institutional vulnerability. This lack of trust prevents communication that would be beneficial to both the people and the national park. Emilie also found that people in Loango have an understanding of sustainability that shares common ground with Western conservation principles. However, farmers cite crop-foraging as the major reason for disliking protected areas and conservation, and these sentiments can result in unobtrusive subversion of conservation efforts, such as feigning ignorance of known conservation laws and legal processes to oppose the imposition of conservation strategies while avoiding direct confrontation (Fairet 2012). In so doing, people can continue to perform illegal activities in good faith, reflecting findings at other sites (De Boer and Baquete 1998; Holmes 2007; Webber *et al*. 2007).

Next, Emilie surveyed fields systematically to record the frequency of crop-foraging events, the extent of damage and crop loss, and the species responsible. Her data show that the level of crop damage is high and unequally distributed across fields (Fairet 2012). Elephants (*Loxodonta africana*) caused the most crop damage, and crop-foraging by elephants followed rainfall and was higher in fields located near permanent water points, probably as a result of seasonal changes in the availability of water and access to the herbaceous vegetation present in swamps (Fairet *et al*., unpubl. data). Moreover, most fields at the study sites are isolated from each other, creating islands of agricultural land surrounded by wild habitat, and Emilie’s results demonstrate that field isolation renders some fields more vulnerable to crop-foraging than others. These findings are valuable, as most sites where crop-foraging has been studied are located in East and South Africa in areas with high human density and a high degree of deforestation, while Gabon has very low human densities (<0.2 inhabitants/km^2^ at the study site) and extensive forest coverage (70–80% of the country is forested: Laurance *et al*. 2006a; b).

Emilie then investigated the use of individual and institutional mitigation strategies against crop-foraging in Loango, which are important to understand both social and institutional vulnerability to crop-foraging. She assessed the efficacy of the deterrent methods present in fields and investigated the financial cost and affordability of deterrent methods for farmers. She found that farmers use diverse deterrent methods to limit crop-foraging, but none seem effective. This lack of efficacy stems mainly from lack of access to labor that is driven by a rural exodus (Fairet *et al*. 2014) and prevents the successful implementation of deterrents. Existing state mitigation strategies are inadequate and ineffective. Ethnography and interviews revealed that people in Loango resent and resist current conservation practices that exclude them, threaten local environmental entitlement, and hinder farmers’ ability to protect themselves against crop-foraging animals. This lack of trust in local authorities and the state makes farmers unwilling to use the institutional pathways at their disposal, increasing institutional vulnerability while preventing adaptive management of crop-foraging (Fairet 2012).

Finally, Emilie used ethnography to investigate the social causes of vulnerability to crop-foraging and the consequences of crop-foraging for farmers’ livelihoods. She explored how crop-foraging, and the need to prevent it, affect farmers’ livelihoods and their ability to prevent further crop damage. She investigated the coping strategies farmers employ to limit the negative effects of crop-foraging, and whether these strategies are successful, providing an overview of social vulnerability to crop-foraging in Loango. Her findings suggest that crop-foraging acts as an additional burden on already vulnerable and marginalized communities in Loango (Fairet 2012). Crop-foraging leads to loss of food and income, which can bring some households to the brink of a subsistence crisis while limiting the availability of cash needed to farm and mitigate crop-foraging. Crop-foraging also induces health problems both directly and indirectly, by increasing the risk of injury and by the increased exposure to mosquito-borne disease, stress, and lack of sleep. Crop-foraging prevents farmers from using fields to ensure access to food and income that act as a safety net during times of unemployment or at retirement. Finally, crop-foraging threatens the farmer’s role within the family and the community. In summary, crop-foraging favors a negative spiral of increased vulnerability to poverty and to crop-foraging itself (Fairet 2012).

Many of Emilie’s results are comparable to those of other studies of crop-foraging (Hill 2000; Kaswamila *et al*. 2007; Lahm 1996; Naughton-Treves 1997; Ogra 2009; Osborn and Parker 2002), which suggests that the structure of her integrated analysis is correct. However, her interdisciplinary approach allowed her to investigate how each domain interacts with others in more depth than previous studies. For example, she found that social vulnerability (e.g., rural exodus and poverty) affects biophysical vulnerability (e.g., animals are not deterred by human disturbance as in the past). She showed that demographic, sociocultural, and political changes have a substantial effect on farmers’ abilities to mitigate and cope with crop-foraging, an effect that may be stronger than changes in animal density. She also showed that the poor quality of stakeholder interactions in Loango affects farmers’ abilities to use existing legal pathways that could provide immediate relief, such as compensation. Finally, she showed that isolation, in both its geographical and social forms, is the dominant force driving vulnerability to crop-foraging in Loango.

Social and institutional vulnerabilities are often overlooked in analyses of crop-foraging and in conservation more generally. However, the combination of three approaches—social, biophysical and institutional—in this study tells us far more about vulnerability to crop-foraging than any of these approaches could have done separately. Moreover, the combination of social science (social and institutional vulnerability) and natural science (biophysical vulnerability) allowed a holistic view of the problem. Emilie used this integrated framework to provide recommendations for the management of crop-foraging in Gabon protected areas network that were scientifically sound and socially appropriate (Fairet 2012).

## Case 2: Wildlife Tourism and Conservation: An Interdisciplinary Evaluation of Western Lowland Gorilla Ecotourism in Dzanga–Sangha Reserve, Central African Republic (Kathryn Shutt)

Wildlife tourism has the potential to produce revenue that can be used for conservation as well as to promote public awareness of conservation issues (Dawson 2001; Mehlman 2008; Wilkie *et al*. 2001). Gorilla tourism is one of the best-known forms of wildlife tourism, and tourism and research based on habituated gorillas are promoted as one of the best means of conserving gorillas and their habitats (Butynski and Kalina 1998; Todd 2008; Weber and Vedder 2002; Williamson and Macfie 2010). The International Union for Conservation of Nature has produced guidelines designed to mitigate the negative effects of tourism on great apes, based on experience from great ape tourism sites (Williamson and Macfie 2010). However, Kathryn’s experience with great ape habituation projects led her to realize that few data are available to assess the balance of risks to gorillas against conservation benefits offered by gorilla ecotourism projects (Jones-Engel and Engel 2006; Klailova *et al*. 2010; Travis *et al*. 2008). For her doctoral research, Kathryn based herself in the Dzanga–Sangha Gorilla Habituation and Ecotourism Project in the Central African Republic to provide an integrated understanding of the factors that influence human–gorilla interactions and their consequences for individual gorillas and for gorilla conservation. The complex interplay of human–human and human–animal interactions in this arena required an interdisciplinary approach. In collaboration with the World Wildlife Fund and partners at the field site, Kathryn designed and applied a combination of social and biological science methods, including: participating in the life of the project, for example, acting as a tourist guide when needed; semistructured interviews; interviewer-administered questionnaires; behavioral observations; and noninvasive measures of glucocorticoids and parasite infection in gorillas.

Research on human–wildlife relations in the context of ecotourism has been criticized for paying little or no attention to the ways in which people, including tourists, construct nature (Russell and Ankenman 1996). Studies of the human experience of the encounters with wildlife are important to ensure that the visitors’ experiences are beneficial to wildlife conservation (Schänzel and McIntosh 2000). Kathryn’s social science research methods were designed to explore the types of tourists engaging in Western lowland gorilla tourism, their constructions of gorillas, their motivations to visit gorillas in the wild, their reactions to their encounters with gorillas, and the effect these encounters have on tourists. Three major themes emerged from her ethnographic data. First, perceptions of wild gorillas as being like us (humans) increase the attraction of gorilla encounters. People seek proximity to wild gorillas because they perceive gorillas as being “human-like,” yet mysterious and rare. In line with other studies of wildlife tourists’ motivations (Curtin 2010a; Montag *et al*. 2005; Muloin 1998; Orams 2000), tourists particularly value close proximity to, and eye contact with, gorillas, because this contact stimulates feelings of emotional connection with a wild animal. However, habituation is designed to reduce animal reactions to visitors, with the ideal result being that animals show no reaction to human observers. Visitors are greatly disappointed when habituated gorillas ignore them.

A second major theme related to the rarity and “authenticity” of the experience. For many tourists, the lack of mass tourism at the study site fits their notions of the site as the “real, pristine, Africa” and bolsters constructions of themselves as different from regular or mass tourists. These perceptions of authenticity and value also fit with previous descriptions of wildlife tourism (Curtin 2010a, b; Montag *et al*. 2005; Russell and Ankenman 1996), where the perceived value of an experience decreases as the numbers of other people it is shared with increases (Urry 1990). A strong contrast between negative constructions of zoo gorillas and more positive notions of wild gorillas is important in tourists’ perceptions of an authentic wildlife experience. However, tourists in this setting know very little about the process of habituation that allowed them to visit wild gorillas. This unawareness of habituation seemingly perpetuates a perception of the habituated gorilla as “gentle” and “tranquil” and may influence tourist behavior toward animals that can, in fact, be dangerous to humans.

Photography emerged as a third major theme in the gorilla tourism experience. Tourists take photographs as evidence of their experience, and of particular moments within it. Tourists particularly value instances where they can be captured in the same frame with a gorilla as evidence of their close proximity to the accepting “gentle beast”. Pleasure is linked to success in capturing images in a manner consistent with descriptions of ocular consumption (Lemelin 2006). For other tourists, however, photography is a burden, a source of anxiety and a distraction from their experience. As in other studies of wildlife tourism, photography disrupts both human–human and human–wildlife interactions (Knight and Cole 1995; Lott 1992; Roe *et al*. 1997; Russell and Ankenman 1996). Efforts to capture images frequently result in intrusive behavior and negative interactions with staff and other tourists. Most importantly, photography emerged as the single greatest cause of tourists approaching too close to gorillas and ignoring other safety regulations, leading to aggressive reactions from the gorillas (see also Klailova *et al*. 2010).

Habituation allows humans to observe wildlife closely (Knight 2009). However, behavioral observations suggest that habituation is highly stressful for the animals concerned (Butynski and Kalina 1998; Rose and Rankin 2001). As part of the biological aspect of her interdisciplinary project, Kathryn examined physiological stress in gorillas at Dzanga–Sangha. She found that unhabituated gorillas that are not involved in wildlife tourism activities have lower mean fecal glucocorticoid metabolite (FGCM) levels than gorilla groups involved in tourism and a group undergoing habituation (Shutt *et al*. 2014). These results are consistent with studies of other species that report higher glucocorticoid levels in animals exposed to habituation processes and subsequent tourism compared with those that are not (Barja *et al*. 2007; Behie *et al*. 2010; Turner 2001). Moreover, the gorilla group that was undergoing habituation had higher FGCM levels than all other groups, including habituated groups and unhabituated animals, suggesting that the process of habituation is physiologically stressful for gorillas, but that habituation reduces this response. Finally, FGCM levels in habituated groups were significantly associated with increasing frequency of violation of the 7 m distance rule by observers, suggesting that some elements of human–gorilla contact still elicit a physiological stress response even in habituated gorillas.

The potential for stress to increase the risk of disease transmission and pathogenesis is a vitally important, but previously unmeasured, potential influence of habituation and tourism on wildlife species. In collaboration with parasitologists, Kathryn found a positive association between FGCM levels and parasite infection that may reflect hormonal suppression of the immune system in gorillas with higher FGCM levels (Shutt 2014). These findings are in line with studies of other primates, showing that increased FGCM levels are associated with increased measures of parasite infection (male chimpanzees, *Pan troglodytes*: Muehlenbein 2006; mandrills, *Mandrillus sphinx*: Setchell *et al*. 2010; red-fronted lemurs, *Eulemur fulvus rufus*: Clough *et al*. 2009).

Once gorillas are habituated to human presence, ecotourism and research activities bring them into daily contact with various groups of people, such as tourists, local staff, and researchers. Great apes are susceptible to human diseases, as a result of their phylogenetic closeness to humans (Köndgen *et al*. 2008; Litchfield 2009). The lack of adequate control of this risk is clearly demonstrated by many photographs and videos showing humans in close proximity to, and deliberately touching, gorillas (Butynski and Kalina 1998) and a study of mountain gorilla tourism, which showed that the mean proximity between humans and gorillas was only 2.8 m (Sandbrook and Semple 2006). In line with these studies, Kathryn found that tourists and other visitors violate safety regulations at Dzanga–Sangha (Butynski and Kalina 1998; Sandbrook and Semple 2006). For example, humans broke the minimum of 7 m proximity rule frequently during visits to the gorillas, increasing the likelihood of human–gorilla disease transmission as a result of close proximity. This occurred most frequently during tourist visits and the rule was broken most frequently by trackers leading the tourist group.

Kathryn explored sociocultural, epidemiological, and management aspects of human interactions with gorillas to identify when, how, and why management regulations that protect gorillas from close interactions with humans fail and the implications for the risk of disease transmission from humans to gorillas. She found that the trackers had a poor awareness of the 7 m rule and had difficulty in identifying this distance in the forest. Moreover, cultural hierarchies between the guides and trackers meant great pressure was placed on the trackers to provide consistent and close observation of gorillas to please tourists. Overall, local staff, specifically the trackers, emerged as a much greater health risk to gorillas than tourists, as a result of their generally poorer access to health care, health education, and the comparatively large amount of time they spend in close contact with gorillas. Both tourists and project staff present further sources of disease transmission when they visit local villages and are exposed to young children and domestic livestock shortly before visiting or working with gorillas and by not changing their clothing and footwear before visiting gorillas (Whittier 2010).

Interrogation of Kathryn’s ethnographic data found that, in general, tourists were poorly informed about habituation and the potential risks to gorillas, although they expressed a keen interest in the topic. Tourists reported that their lack of knowledge of the risks reduced their ability and motivation to address them. Thus, rather than simply highlighting nonadherence, ethnography revealed the reasons why adherence is poor, and thus how this can be improved. Kathryn also found that tourists who were unwell visited gorillas, posing a risk of disease transmission. She linked this to management conditions at the time of the study, which precluded effective monitoring of tourists’ health status before arrival at the tracking camps. Tourists said that they wished to support efforts to protect gorillas and would follow regulations, including declaration of illness, updating vaccinations, and providing evidence of vaccination, if they understood why this was necessary. Similarly, tourists said they would be happy to wear facemasks if the risk of transmitting disease to gorillas was explained. This analysis led to simple recommendations for improving management of the project (Shutt 2014). Finally, approximately a quarter of tourists were not well informed about disease risk to themselves, and had inadequate vaccinations, reflecting other findings showing tourists’ low adherence to travel health advice and inaccurate perceptions of disease risk (Lopez-Velez and Bayas 2007; Muehlenbein and Ancrenaz 2009; Piyaphanee *et al*. 2009). The same tourists showed inaccurate perceptions of risks to gorilla health (Shutt 2014).

**Fig. 2:**
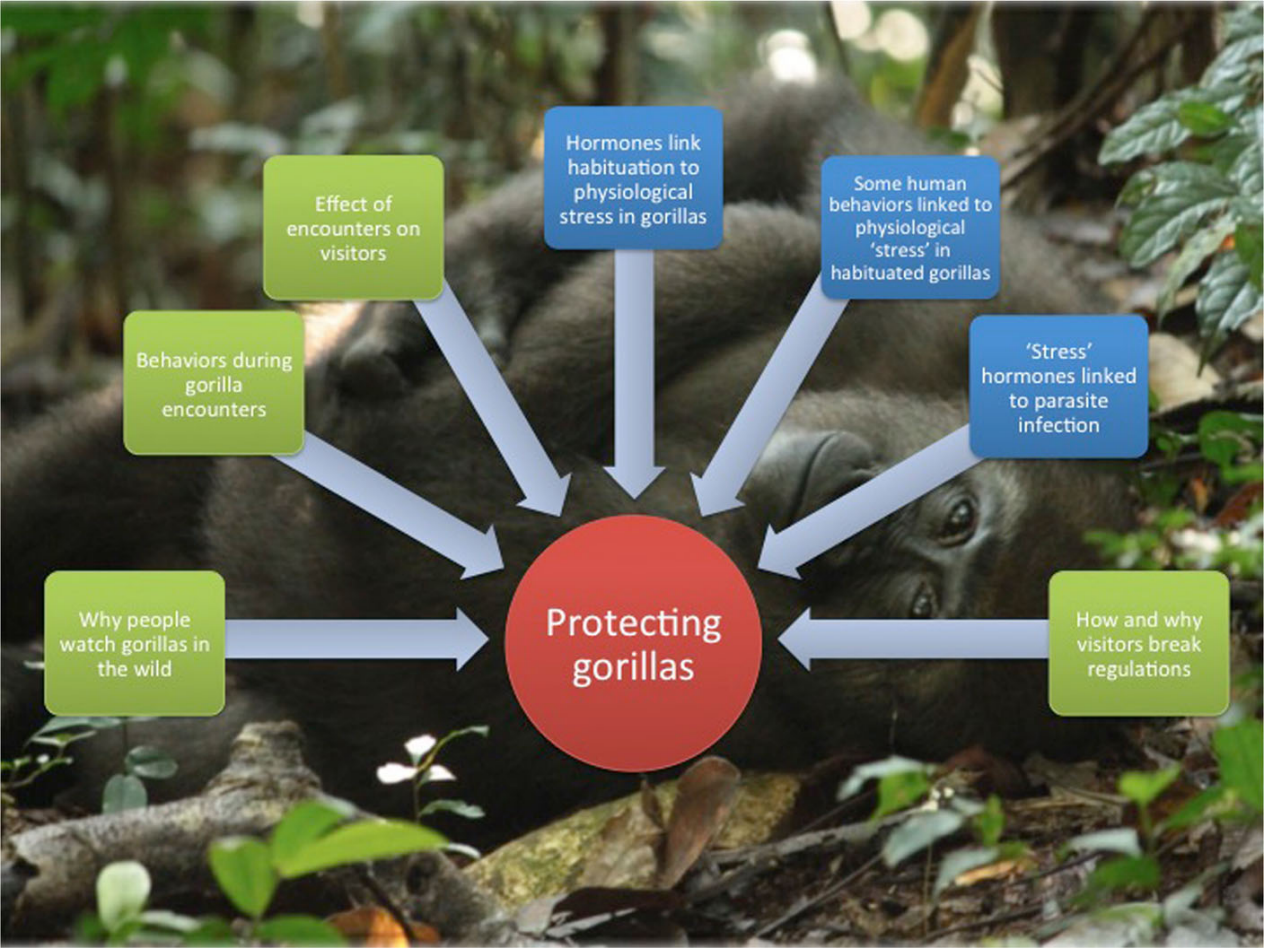
Wildlife tourism and conservation: An interdisciplinary evaluation of Western lowland gorilla ecotourism in Dzanga–Sangha Reserve, Central African Republic (Kathryn Shutt). A young Western lowland gorilla habituated for tourism. Red indicates the overall aim of the study; green, use of social science methods; blue, use of natural science methods.

The combination of biological and social methods in this study reveals far more than either approach alone (Fig. 2). The outcomes of this interdisciplinary risk assessment are a set of recommendations for the specific site (Shutt 2014), many of which can be applied to other wildlife tourism settings, and will help to maximize the potential for projects to be beneficial, low-impact, and sustainable conservation solutions.

## Case 3: Including People in Primate Conservation: Shepherds and Barbary Macaques in Bouhachem forest, Northern Morocco (Siân Waters)

Conservation projects for individual species are more effective if they incorporate local people’s knowledge and perceptions of that species (Horwich and Lyon 2007; Measham and Lumbasi 2013). For example, a participatory approach to African elephant conservation led to the successful inclusion of local people in conservation and research activities in the Samburu district of Kenya (Kuriyan 2002). However, the effective inclusion of local people in conservation initiatives is a complex undertaking. Poor relationships between the various actors involved, along with imbalances in power relationships, have caused many conservation strategies to fail (Geoghegan 2009; Russell and Harshbarger 2003), with Emilie’s study documenting one example of a lack of trust and engagement between local people and officials (Fairet 2012). Siân runs Barbary Macaque Awareness and Conservation, a conservation project for the Endangered Barbary macaque (*Macaca sylvanus*) in Bouhachem forest, Northern Morocco. For her doctoral research, Siân was thus able to practice participatory action research, a style of research that entails sustained engagement and building of trust with participants in a project. Siân’s aim was to investigate how effective ethnographic methods could be toward creating a conservation strategy that involves local people and draws on their extensive ecological knowledge.

Conservationists need to examine how historical, political, and sociocultural factors have influenced local people’s perceptions of, and interactions with, outside agencies (Brosius *et al*. 1998) to avoid the use of conservation activities that alienate a disempowered population (Fairet 2012). As Emilie’s project demonstrated, local people’s interactions with their environment involve diverse social, cultural, and political perspectives that may not be immediately obvious to an incoming conservationist (Wolverton *et al*. 2014). For example, many countries have a history of forceful imposition of conservation by colonial powers (Redford 2011), or by the state, marginalizing local people (Benjaminsen *et al*. 2013). Siân began by investigating and reflecting on the historical, political, and sociocultural context of her study area. Her findings suggest that local people’s experiences with outside agencies have been characterized by a history of exclusion from decision making about the forest they use to sustain their livelihoods, as well as negative discrimination by urban dwellers. The history of marginalization suggested that a conservation strategy concentrating only on the macaques would have alienated local people further. Thus, the most important conservation action of her study became her frequent, regular, and open contact with the shepherds who share the forest with macaques.

People are more likely to participate in resource management or conservation initiatives when their knowledge is sought, incorporated, and built on than otherwise (Graham *et al*. 2011; Kuriyan 2002; McNeely and Scherr 2003; Pretty and Smith 2004; Young *et al*. 2013), highlighting the importance of consistent and regular contact with, and commitment to, local people by conservationists. Shepherds are key players in the case of the Barbary macaques of Bouhachem and have detailed knowledge of where macaques live in the landscape. Siân integrated her own “conventional” scientific knowledge with the shepherds’ local ecological knowledge of macaques to co-produce information about the population status of Barbary macaques in Bouhachem. Such knowledge co-production is “a process where knowledge is or can be produced through interaction with others, possibly with people with different perspectives and backgrounds, through cooperative endeavours and mutual learning” (Fazey *et al*. 2012: 20) and necessitates the building of trust between participants (Grimwood *et al*. 2012; Sillitoe 2010). This aspect of the study achieved two aims. First, as in other studies (Gilchrist *et al*. 2005; Turvey *et al*. 2013, 2014; Ziembicki *et al*. 2013), incorporating local ecological knowledge into her study improved Siân’s understanding of macaque distribution and abundance. Second, regular personal contact between the conservation team and shepherds, as well as the inclusion of shepherds in the macaque population survey, had enormous benefits in terms of establishing close relationships with a group of people who regularly use Barbary macaque habitat. It inspired the shepherds to feel “ownership” of both the research and the macaques, much like fishers involved in research to identify the scale of predation by seals (*Halichoerus grypus* and *Phoca vitulina*) on Atlantic salmon (*Salmo salar*) in Scotland (Young *et al*. 2010). Siân continues to employ integrated surveys for other Barbary macaque populations in the region.

Siân also used ethnography to examine how people viewed both wild and domestic animals that use the forest, including macaques. She found that shepherds’ relationships with goats (*Capra hircus*) and the largest wild canid in the forest, the African wolf (*Canis lupus lupaster*), are relatively uncomplicated, relating directly to goats as prey and wolves as predators. The shepherds’ relationship with their dogs (*Canis familiaris*) is more ambiguous. Shepherds appear to resent the presence of dogs, but accept their role as a protector of livestock. The position of dogs in Bouhachem society mirrors the position of dogs elsewhere, symbolically existing “between the human and non-human worlds” (Serpell 1995: 254). When it comes to macaques, the shepherds’ ontology is influenced by the Islamic view of primates as degraded humans, but is not entirely explained by this. Siân’s preliminary contacts with groups of shepherds led her to think that the macaques had little intrinsic value for local people. However, on interviewing individual shepherds, she discovered that some possessed detailed knowledge of macaque movements and diet, which contrasted with the less detailed knowledge of shepherds who professed to have no interest in the macaques or other wildlife. However, the knowledgeable individuals concealed their interest in macaques for fear of ridicule by their peers. Over time, the presence of the conservation team made it socially acceptable to discuss macaques without fear of being mocked or shamed.

The ambiguous position of the Barbary macaque in shepherd ontology is embodied in shepherds’ physical interactions with macaques in agricultural and forest spaces. Barbary macaques forage on crops during lean seasons in the study area. Siân found that local people punish crop-foraging macaques, attempting to “teach them a lesson” by alienating them from their social group. The shepherds know it is unusual to see a macaque alone in the forest and their attempts to deliberately alienate a macaque from its group may reflect their own fear of social exclusion should they behave in an uninhibited, macaque-like way and thus transgress the social conventions of their own society. Although people kill other crop-foraging species, they do not kill macaques in this context. However, young shepherds use their dogs to hunt, capture, and kill Barbary macaques in the forest at other times. Siân suggests that the shepherds’ behavior toward the macaques changes with a shepherd’s age and is related to the shepherds’ position in village society.

Local people may not share the intrinsic value placed on wildlife by conservationists and do not view its protection as a priority (Adams and Hulme 2001), but such views are not static. Siân’s inclusion of shepherds in research activities and her understanding of the reasons for their negative behavior toward the macaques enabled her to foster a change in their attitudes from macaque hunting toward conservation. Over time, Siân’s informants voluntarily established themselves as role models to encourage other shepherds to stop macaque hunting. They also acted to prevent illegal capture and trade of “their” macaques by outsiders. Shepherds spontaneously reported observations of macaques and of incidents involving macaques to members of the conservation team during open-ended interviews or informal chats after the more formal semistructured interviews had taken place. This contact with the conservation team seemed important to the shepherds as a way to express interest in the macaques. These observations are in line with literature suggesting that social mechanisms are more effective at inspiring positive conservation action than state intervention, which may have the opposite effect to that required for species conservation (Bell *et al*. 2008; Carss *et al*. 2009; Fairet 2012; Goldman *et al*. 2013). A similar understanding of Maasai tradition has been used to discourage killing of lions (*Panthera leo*) in Kenya (Hazzah *et al*. 2014).

Effective communication with local people is fundamental to successful conservation (Bickford *et al*. 2012). This is particularly important in cases where local and scientific knowledge conflict (Dowsley and Wenzel 2008). Conservationists often assume that empirical findings will be sufficient to persuade local people to change people’s opinions or behaviors, but this may not be the case (Redpath *et al*. 2013; Saunders 2011). Moreover, the communication of findings that oppose local beliefs can cause alienation and conflict (Peterson *et al*. 2013; Redpath *et al*. 2013). During her study, Siân was faced with an example of such conflicting information. Dogs prey on macaques in the forest, and shepherds in Bouhachem also reported losing livestock to a pack of feral dogs that roamed the forest. During her surveys, Siân discovered that the purportedly feral dog pack was in fact made up of domestic dogs owned by people living in the study villages. Aware that this was potentially difficult information to communicate directly, Siân instigated a rabies vaccination program for village dogs. The program used colored collars for vaccinated dogs, allowing shepherds to identify the forest dogs. Thus, Siân and her team communicated the message that the “feral” dogs were owned by villagers without needing to challenge local people’s understandings directly. At the same time, the intervention also reassured local people that their health and well-being are important to the conservation project.

**Fig. 3:**
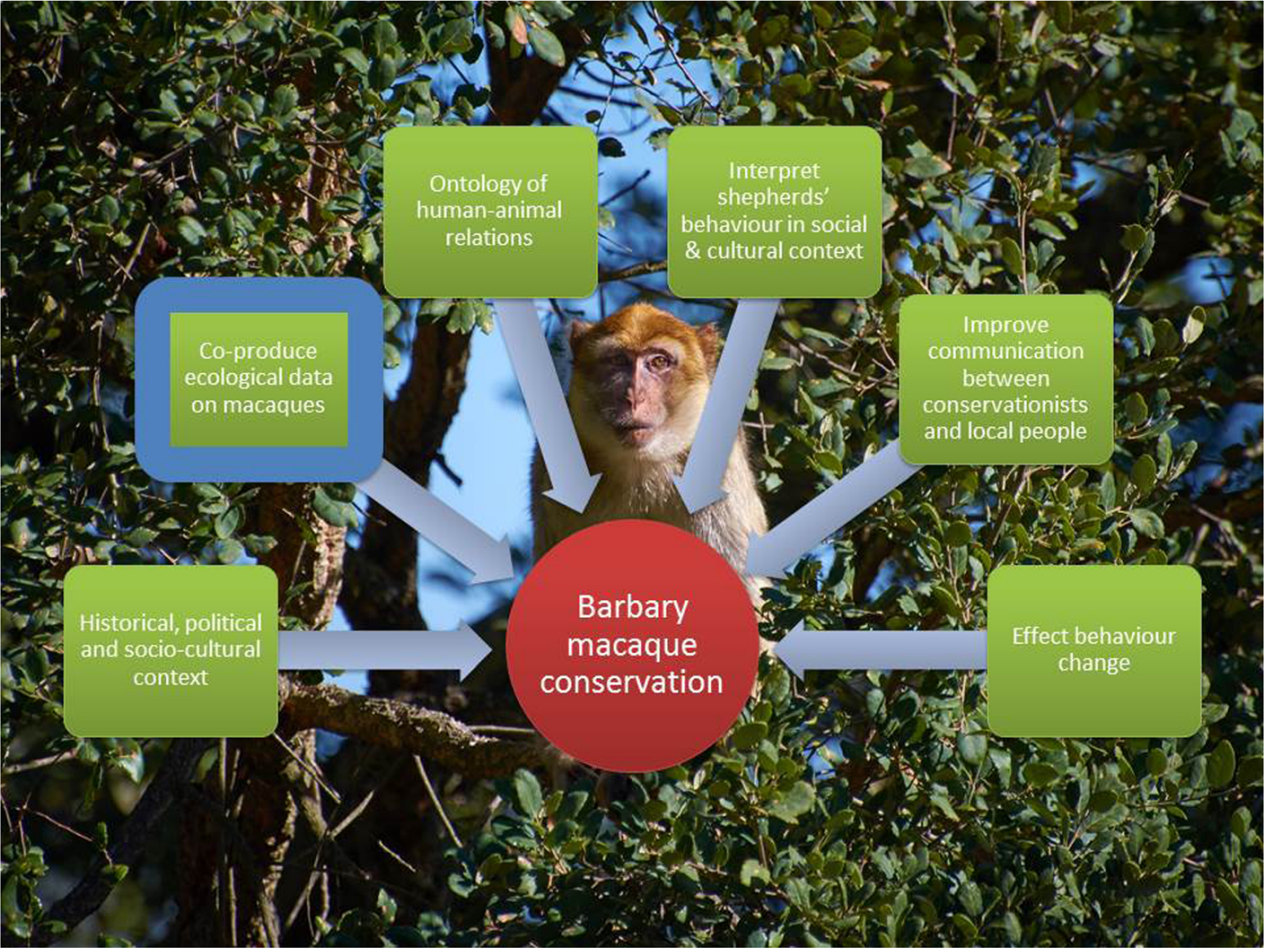
Including people in primate conservation: Shepherds and Barbary macaques in Bouhachem forest, Northern Morocco (Siân Waters). A Barbary macaque in Bouhachem Forest (photo by Lucy Radford). Red indicates the overall aim of the study; green, use of social science methods; blue, use of natural science methods.

Overall, Siân’s study illustrates how the collection and analysis of ethnographic data provide an important, and probably essential, dimension to successful conservation practice. In the end, her project was almost entirely ethnographic (Fig. 3) and fundamental to her understanding of the social and cultural factors underlying shepherds’ relations with macaques. In the Bouhachem villages, a one-dimensional conservation strategy concentrating only on the macaques would have alienated local people. Instead, by making people central to the project, the conservation team found that shepherds took the lead in effecting a change in their peer group’s behavior toward the macaques.

## Conclusions: Bridging Boundaries of Understanding

The insights obtained from ethnographic data in these three studies show that this type of data collection and analysis is vital to our understanding of conservation problems, supporting earlier calls for the integration of qualitative methods in such studies (Goldman *et al*. 2010; Moon and Blackman 2014; Redford 2011; Satterfield *et al*. 2013), and reflecting the findings of ethnoprimatological studies of relations between humans and other primates (Fuentes 2012; Fuentes and Hockings 2010; Hill and Webber 2010; Malone *et al*. 2014; Nekaris *et al*. 2010). The qualitative ethnographic approach in each of our case studies provides a far richer understanding of the context of the specific social context and conservation issues than a quantitative approach could hope to, illustrating the need to understand and acknowledge different value systems, histories, and cultural viewpoints in the quest for greater understanding of the factors characterizing complicated relationships between people and animals. Emilie’s project emphasizes the advantages of employing participant observation (Drury *et al*. 2011). The same informants who were open during informal conversations, even when Emilie was explicitly taking notes and/or recording them, avoided discussing sensitive issues when she made an appointment to conduct what felt like an official interview. Her participant observation was also extremely valuable in highlighting contradictions between what people said and did, and in what people said at different times during the study. The qualitative aspects of Kathryn’s study shed far more light on why people visit gorillas, their experiences, and why they break the rules than a simple questionnaire could do. Siân doubts that she would have discovered the widespread hunting of macaques by shepherds by using a questionnaire. Moreover, she is convinced a conservation project concentrating only on the macaques would have alienated local people in her study area. The analysis of ethnographic data also requires a reflexive awareness of the author’s own position within a research project that can greatly assist conservation practice. For example, Emilie’s understanding of the difficulties of villagers’ daily lives challenged her conception of conservation outcomes and success, Kathryn was able to glean a wholly different perspective of tourist–gorilla interactions whilst herself operating as a guide and medium of the experience and Siân found reflection on ethnographic data was extremely important in evaluating how to act or, indeed, whether to act.

The three projects reviewed here highlight the benefits of an integrated biosocial approach to issues in conservation. They go beyond conducting quantitative and qualitative analysis in parallel (i.e., multidisciplinary research), to integrate the datasets, producing results that would not otherwise have come to light (i.e., interdisciplinary research). Such interdisciplinary projects are challenging to conduct. Many authors have described the boundaries of understanding and communication between academic disciplines with regard to conservation (Campbell 2005; Heberlein 1988; Lowe *et al*. 2009; Moon and Blackman 2014). Barriers include disciplinary chauvinism and territoriality, tokenism, and overdominance of any one perspective (review in Newing 2010). Research that transcends conventional academic boundaries requires the openness and flexibility to move beyond one’s comfort zone to understand and acknowledge the legitimacy of “other” knowledges. It requires deep engagement with profoundly different, and unsettling, ways of thinking, collecting information, analyzing, and writing. Mastering a new discipline is time consuming and challenging (particularly given the time constraints of a UK PhD). Interdisciplinary research is harder to conduct, write about, evaluate, and publish than research in a single discipline (Charnley and Durham 2010; Strang and McLeish 2015). Moreover, the use of qualitative as opposed to quantitative data remains an obstacle when integrating social science into a conservation program because qualitative data do not readily provide measureable (and auditable) outcomes. The economic, and sometimes logistical, constraints of collecting reliable scientific data, along with a general desire for numbers in Western culture, result in pressure on scientists to provide these numbers even where the methods are imprecise (Mehlman 2008). This pressure is also felt by conservation organizations that are pressured to provide such data for donors (Goldman *et al*. 2013). Nevertheless, our experiences confirm that integrating disciplines is important for conservation.

Interdisciplinary research can provide detailed understanding of the context of a conservation problem and make recommendations for improvement (Robinson 2006). However, the power of the researchers to implement such recommendations varies. In Emilie’s study, we made recommendations to nongovernmental organizations and the government, which are responsible for management decisions. In Kathryn’s study, the managers of the gorilla tourism project requested the study, were involved in the project from its inception, and were keen to implement the recommendations. Other tourism projects have also adopted the recommendations. In Siân’s case, she leads the conservation project, meaning that she can implement the findings herself. Where researchers cannot implement findings themselves, an interdisciplinary approach can also help to understand managers’ decisions as to whether to implement recommendations.

The case studies we present serve to highlight the need for researchers to become skilled at bridging disciplinary boundaries to provide a better understanding of the complexity of the context in which conservation occurs (Chan *et al*. 2007; Fox *et al*. 2006; Pretty 2011). The methods we advocate are particularly relevant for the study of primates in human-dominated landscapes, but also apply more broadly to primate conservation in any landscape. We hope that our experiences will encourage primatologists with a life sciences background to engage deeply with social anthropology to illuminate issues in primate conservation.
